# Sentiment analysis of Indonesian datasets based on a hybrid deep-learning strategy

**DOI:** 10.1186/s40537-023-00782-9

**Published:** 2023-05-29

**Authors:** Chih-Hsueh Lin, Ulin Nuha

**Affiliations:** Department of Electronic Engineering, National Kaohsiung University of Science and Technology, Kaohsiung, 80778 Taiwan

**Keywords:** Sentiment analysis, Hybrid model, Text representation, BERT, Classifier models

## Abstract

Various attempts have been conducted to improve the performance of text-based sentiment analysis. These significant attempts have focused on text representation and model classifiers. This paper introduced a hybrid model based on the text representation and the classifier models, to address sentiment classification with various topics. The combination of BERT and a distilled version of BERT (DistilBERT) was selected in the representative vectors of the input sentences, while the combination of long short-term memory and temporal convolutional networks was taken to enhance the proposed model in understanding the semantics and context of each word. The experiment results showed that the proposed model outperformed various counterpart schemes in considered metrics. The reliability of the proposed model was confirmed in a mixed dataset containing nine topics.

## Introduction

In recent years, the progression of classification tasks in machine learning, including natural language processing (NLP), has grown rapidly. One of the most concerning classification tasks in the study of NLP is sentiment analysis. This analysis can reveal public opinion related to an issue in text-based messages [1]. Such an observation of public opinion is valuable to evaluate the products and services of an institution or corporation. Public sentiment regarding an issue is generally categorized into positive, negative, or neutral responses. Various existing studies on sentiment analysis have focused on detecting a subjective sentiment on a particular topic, such as hotel reviews, mobile app reviews, public opinion related to current issues on Twitter, or others [2–4]. In addition, the existing sentiment analysis studies mostly investigate issues using English-language data.

Based on level, sentiment analysis can be categorized and conducted in diverse levels, they are document, sentence, and aspect levels [5]. Sentiment analysis based on document aspect determines the sentiment polarity of a long document such as news articles. For the sentence level, this analyzes the sentiment of texts in short documents such as news titles or comments on social media platforms such as Twitter and Instagram. To extract a specific target of features in a review, the aspect-based sentiment level is commonly selected. In this paper, our work determines the sentiment analysis in a short document or sentences. The challenges faced in the classification of short documents are their scant and irregular structures [6]. Various techniques in sentiment classification, including traditional methods, machine learning, and hybrid models, have been proposed by researchers to address several challenges.

The crucial component of text-based deep learning is text representation, which is used to make computers understand text data in a numerical form. The latest prominent text representation technique is bidirectional encoder representations from transformers (BERT), which has achieved optimal results in multi-task downstream of the NLP study. BERT is a pre-trained language model that can be applied in diverse tasks such as text classification, answering questions, and text summarization by performing a fine-tuning process. The BERT architecture consists of stacked layers of a bidirectional transformer encoder and utilizes unsupervised tasks in the pre-training process [7]. To overcome shortcomings in the standard BERT method, several forms of BERT have been developed, such as the robustly optimized BERT pre-training approach (RoBERTa) and a distilled version of BERT (DistilBERT) [8].

In previous studies, a hybrid of a classifier model for sentiment classification tasks was introduced to attain better accuracy compared to the single model. Several studies have presented hybrid approaches based on long short-term memory (LSTM), convolutional neural networks (CNNs), and support vector machines (SVMs) to better understand the information and relationships of text data [9, 10]. Yaseen et al. proposed a hybrid model of document embedding by leveraging the combination of BERT and RoBERTa to grasp word complexity in text passages [11]. This work obtained the final prediction by introducing the weighted average from each text representation.

Considering several text representation models and various deep learning classifiers available for the classification task, a question comes to us. That is whether the hybrid model is introduced on the text representation or the classifier model section to improve the sentiment classification performance. Therefore, this study investigated the impact of hybrid models in sentiment classification analysis on the accuracy of the results. This paper introduced the hybrid approach not only based on document embedding or text representation but also on the classifier model. In addition, the examined data incorporated various topics obtained from several platforms in the Indonesian language. The main contributions of this research were as follows:

1. This study proposed a hybrid classification model of sentiment analysis in the Indonesian language.
2. This study utilized the combined BERT and its variants to train the document embedding vectors during the text vectorization procedure and enhance the reliability of the text representation.
3. This study introduced a hybrid architecture based on bidirectional long-short term memory (Bi-LSTM) and temporal convolutional networks (TCN) to better understand the contextual and semantics features.
4. This work focused on Indonesian sentiment studies using datasets consisting of various topics from several platforms.

The rest of the paper is arranged as follows: “Related work” section conveys a review of literature related to this work; “Methods” section presents the framework of the proposed architecture and methodology, and “Experiments and results” section conveys and discusses the experimental results. Finally, “Conclusion and future work” section concludes the work and provides a direction for future research.

## Related work

The first crucial section in the text classification task is text representation known such as word embedding. In recent years, the most popular text representation methods have been term frequency-inverse document frequency (TF-IDF) and Word2vec. Since the architecture of BERT was introduced, various works have replaced text representation models with the BERT transformer-based model [6, 9]. The remarkable result of BERT, as compared to the previous NLP models, is obtained by using a pre-training process on a massive corpus [12]. Then, various sentiment analysis studies have been proposed using both traditional techniques and machine learning. Sergio [13] proposed BERT for the encoding of text representation in short document classification. The BERT encoder performance was then compared to a number of baseline encoders, i.e., Bag-of-Words (BoW) and TF-IDF. Using the multilayer perceptron (MLP) as the classifier model, the text encoding using BERT obtained the best classification performance. Zheng et al. employed a classifier model with BERT text representation to analyze Chinese sentiments in computer-related course reviews [14]. Then, they proposed the bi-directional gate recurrent unit (Bi-GRU) as the classifier model and achieved superior results compared to the traditional machine learning model. In another study, the combination of RoBERTa and LSTM was introduced to learn the long-distance contextual semantics and classify three sentiment classes [15]. Its work augmented the dataset with GloVe word embedding to prevent oversampling of the minority classes.

In Samadi et al. [16], various transformer-based word embedding models such as BERT and others were combined with various classifier models to classify long documents. The results showed that RoBERTa and funnel transformers with multiple-CNNs as the classifier had excellent performance. Dang et al. [9] introduced a variety of experiments between feature extraction and classifier models. This examined the best hybrid model of sentiment polarity analysis in various huge data. After the experiment stage, the researchers claimed that the hybrid model exploiting SVM attained higher reliability than the model without SVM. Bao et al. [6] recommended BERT-based text representation and a hybrid model of CNN and attention-based Bi-GRU to enhance the capability of classifying the sentiment of short sentences. CNN and attention-based Bi-GRU units were placed in a parallel sequence, and the outputs of each unit were concatenated to obtain the final prediction. Although the results of this proposed model indicated higher accuracy, the difference in accuracy with counterpart schemes was not significant after comparison. A fusion model of classification text incorporating BERT, CNN, and LSTM was presented to better understand the semantic information [17, 18]. Mohbey et al. [19] also proposed CNN and LSTM to increase the prediction accuracy but they exploited the Glove vector in text representation. Another study related to the sentiment classification of film reviews and the Stanford sentiment treebank (SST) was performed employing the RoBERTa model to decompose word vector representations [20]. Bi-GRU was taken to extract the features of the text representation and the attention mechanism following it to assign the addressed features. This model was followed by the softmax activation function to classify the final sentiment prediction. The metrics values in the experimental evaluation indicated good performance. Meena et al. [21] proposed a sentiment classification model by exploiting Keras embedding to generate feature vectorization and CNN to learn the deep information of text data to a final classifier. Table 1 below summarizes some of the previous works.

**Table 1:** Summary of related works in text classification using language modeling

Publication	Technique applied	Claimed outcome	Limitation
[6]	BERT and a hybrid model of CNN and attention-based Bi-GRU	The proposed technique attained the best scores of metrics in the experiment	Trained in a single dataset and English language
[9]	A combination of feature extraction and classifier models in various dataset	Using SVM as a classifier and CNN & LSTM as feature extraction improves the performance result	Longer computation time and trained in English language
[16]	Proposing various pre-trained models for word embedding and classifiers	RoBERTa and funnel transformers with CNNs as the classifier had excellent performance	Get a low accuracy on the LIAR dataset
[17, 18]	A fusion model based on BERT and LSTM-CNN for prediction	The proposed technique obtained the best result of metrics scores	Trained in a single dataset and English language
[19]	Glove vectors and a hybrid model of LSTM-CNN	A hybrid scheme to determine the emotional polarity of tweets with high accuracy	Trained in a single dataset and English language
[20]	Using RoBERTa as text encoder and BiGRU & attention as feature extractor	A pre-training language model is effective for word vector extraction and the addition of attention can improve the accuracy	Get a low accuracy on the SST dataset
[21]	Keras embedding and convolutional neural network	Achieving accuracy levels above 94%	Focused on short sentences with maximum words of 20

In studies on Indonesian sentiment analysis, Antonio et al. performed a study of public opinion on Twitter related to Covid-19 issues [22]. They proposed TF-IDF as the text feature extraction and the stochastic gradient descent (SGD) method to classify the final prediction. Due to the simplicity of the proposed approach, the performance accuracy did not achieve superior prediction accuracy. A sentiment analysis of mobile app reviews was performed using the Indonesian pre-trained BERT model to gain improved context representation [23]. The mined data from Google Play Store website was then given a lexicon-based label. The study also considered score-based reviews on the website and lexicon-based method. The pre-trained BERT model outperformed standard machine learning models such as random forest and SVM in the classification task performance. Fimoza et al. [24] conducted a sentiment analysis of Indonesian film reviews in three polarities. The work exploited the BERT model to better understand the word context. Then, the softmax activation function was selected as the final classifier component. However, the work did not achieve a satisfactory result. Table 2 shows the existing sentiment analysis studies in Indonesian language.

**Table 2:** The existing sentiment analysis studies in Indonesian language

Publication	Technique applied	Claimed outcome	Limitations
[21]	TF-IDF as text vectorization and the stochastic gradient descent (SGD) as the classifier	The proposed technique obtained an accuracy of 81,726%	Not achieving superior accuracy due to its simplicity in the proposed scheme
[22, 23]	Sentiment classification of reviews using BERT and Softmax	Pre-training language model outperforms lexicon-based and review rating schemes	Not achieving superior accuracy due to its simplicity in the proposed scheme

Based on the aforementioned studies, various existing hybrid models have focused on the classifier model. Then, Yaseen et al. [11] designed the BERT model combined with the RoBERTa model to predict lexical complexity. The outputs of each pre-trained language model are followed by the weighted average to get the final prediction. In another study, Briskilal and Subalalitha [25] introduced the combination of the BERT and RoBERTa models to attain deep contextual representation in detecting idiomatic sentences. The final prediction of their work was obtained after implementing a weighted average strategy for the BERT and RoBERTa outputs.

Even though various studies have succeeded in implementing deep neural approaches for sentiment analysis, little research in this field has been conducted in the Indonesian language. In addition, the existing hybrid models have been applied only in text representation or the next stage, i.e., the classifier model. Therefore, this study proposed a general framework that could exploit the hybrid model of text representation and classifier models to improve the accuracy of understanding contextual and semantic features among various data topics.

## Methods

### Data preprocessing

**Fig. 1:**
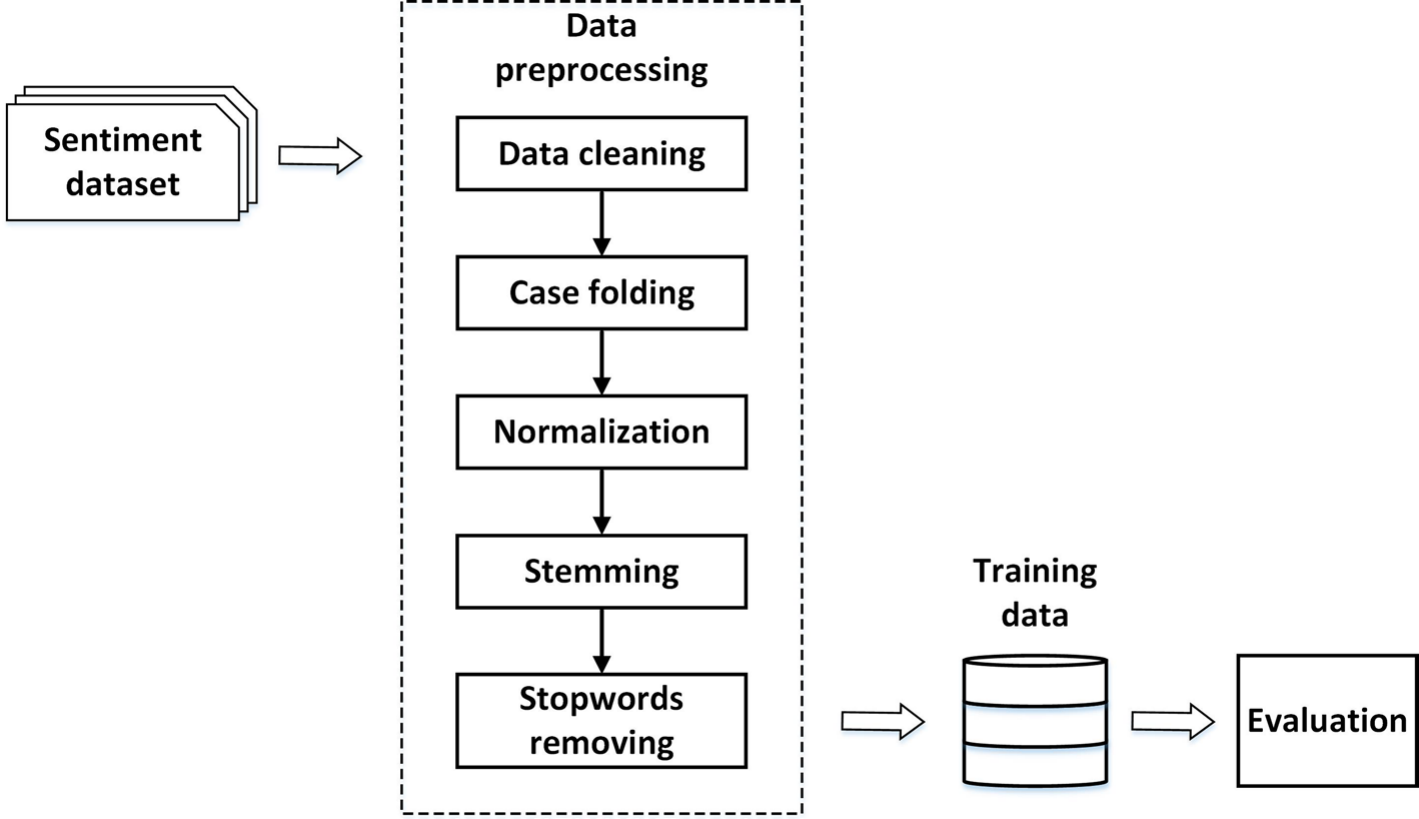
The general preprocessing framework

Since the data used in this study were obtained in the form of raw text, we required proper data to be processed. Therefore, we first performed data preprocessing on the datasets to enhance the text extraction. In addition, such an approach can have a great impact on text processing [26]. The data preprocessing steps, shown in Fig. 1, were as follows:

1. Data cleaning
  Text documents in a dataset mostly contain characters, links, usernames, numbers, symbols, and others that can obstruct the text analysis process. Therefore, we introduced this process to detect unnecessary characters and information, then deleted them.
2. Case folding
  Case folding was employed to change all uppercase letters in each word into lowercase, so as to generalize the dataset in accordance with the requirements [27].
3. Normalization
  Sentence documents mined from social media frequently contain slang words. For example, bokap means “ayah” in Indonesian or “dad” in English. Such slang words can generate more vector dimensions and increase the computation process [28]. It is critical to convert slang into standard words according to their orthography.
4. Stemming
  Stemming is a technique used to modify words by eliminating affixes such as prefixes and suffixes to the root word and therefore shorten the vocabulary space.
5. Stopword removal
  Stopword removal is used to eliminate words that have no much meaning to a sentence in text processing, such as “the”, “also”, and others.

### The proposed model

**Fig. 2:**
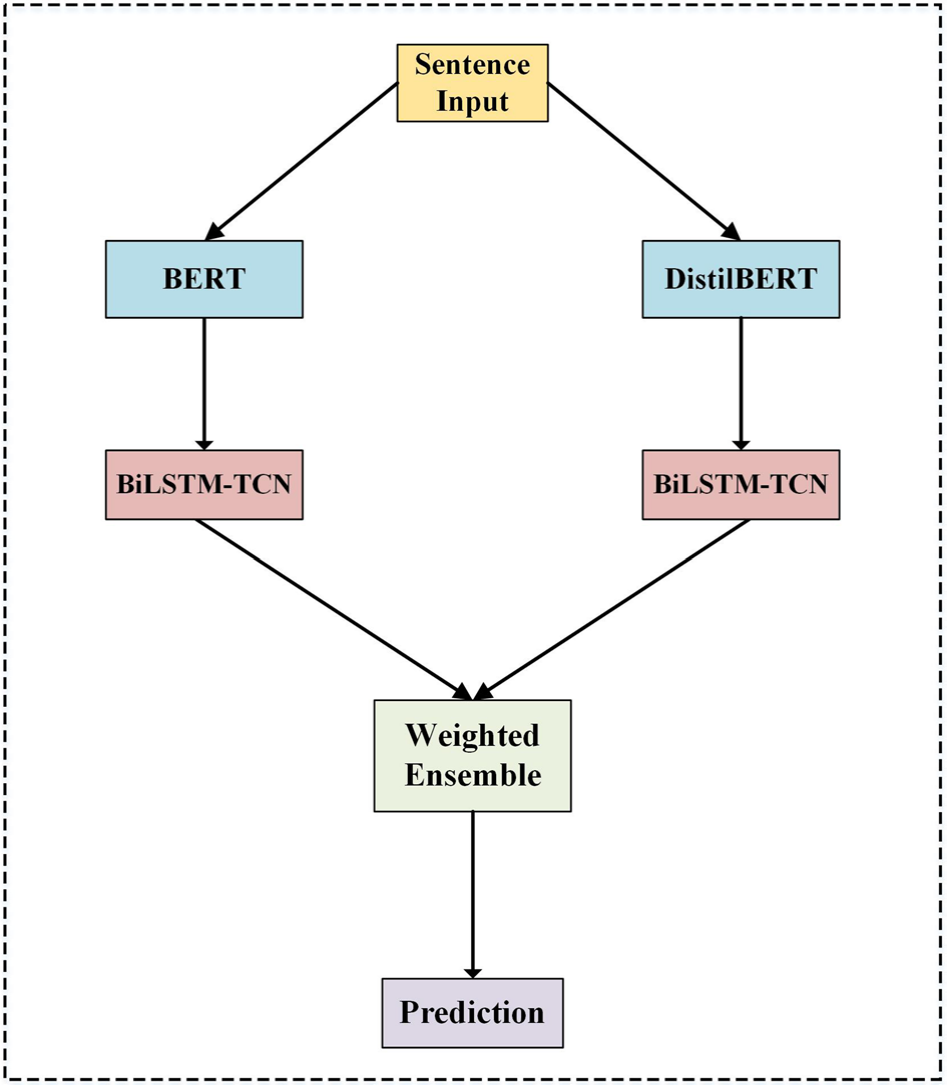
The proposed hybrid strategy

This study proposed a hybrid model based on the text representation and the classifier model. We first took into account the pre-trained language text representation models. For this text representation, we introduced the combination of BERT-large and the DistilBERT-base. Figure 2 depicts the proposed model for the sentiment classification task. Initially, a sentence input is inserted into the text representation to obtain the feature representation vector of BERT and DistilBERT. Here, the selected output of these pre-trained language models is pooler output. The pooler output is the last layer hidden state of the classification token (CLS) after further processed by a linear layer and a tanh activation function. Then, the pooler output is fed to Bi-LSTM and TCN. Both Bi-LSTM and TCN are exploited to improve the prediction of the sentence sentiment. Bi-LSTM is employed to grasp the contextual and temporal features with bidirectional learning in text representation features. Then, TCN is employed to understand the critical local and spatial features of the input sentences. Thus, the proposed model can learn the patterns and sequences of various text information features to achieve a deep understanding of the sentiment. The final stage of the proposed strategy presents a weighted ensemble scheme determining the final prediction by weighting the previous outputs.

### Text representation

Text representation is a critical part of natural language tasks used to represent texts that can be understood by machines. The text representation process has several challenges. Semantic and syntactic issues are crucial challenges faced in delivering text representation [16]. BERT is the current superior method for addressing text representation. This achievement is obtained by training the model in two tasks, i.e., the masked language modeling (MLM) and next sentence prediction (NSP) mechanisms. The MLM mechanism masks a random word in a sentence, and the model then estimates the masked text based on the surrounding masked word to learn the context [29]. For the NSP mechanism, a pair of sentences is introduced to perform binarized next sentence prediction. The NSP mechanism will determine whether the second sentence of two sentences is the pair of the first sentence or not. This learning process can help the pre-trained model remember the relationship between the texts.

**Fig. 3:**
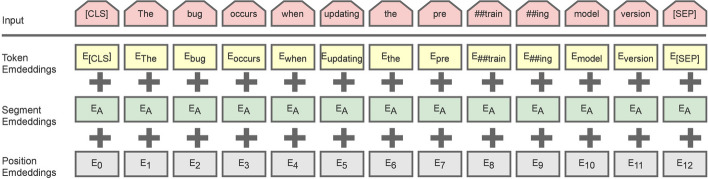
Input representation in the BERT model [30]

In the input representation of BERT, the components consist of token embeddings, segment embeddings, and position embeddings, as shown in Fig. 3. The token embeddings in the BERT architecture consist of word tokens obtained by the WordPiece method, the token embeddings always start with [CLS] token and end with the [SEP] token. Then, the segment embeddings are assigned to denote every token belonging to a certain sentence, and the position embeddings indicate the position of each word in the sentences. An element-wise summation is then performed on these three components. Thus, BERT can handle various down-stream tasks.

Since the emergence of BERT, other variants have been proposed, such as DistilBERT, RoBERTa, and others. As mentioned in the proposed model, we selected the hybrid strategy of BERT and DistilBERT in consideration of the reliability and computation time. The DistilBERT model is a faster and lighter version of the original BERT model that uses a distillation method or compression technique while preserving about 97% of BERT’s performance [31]. This variant removes token-type embeddings to determine which token belongs to which segment. DistilBERT also removes the NSP mechanism during pre-training to create a simpler training process while maintaining performance. The MLM mechanism is still maintained, but dynamic masking is introduced using another masked word in the sentence, in contrast with BERT, which applies static masking.

### The classifier model

**Fig. 4:**
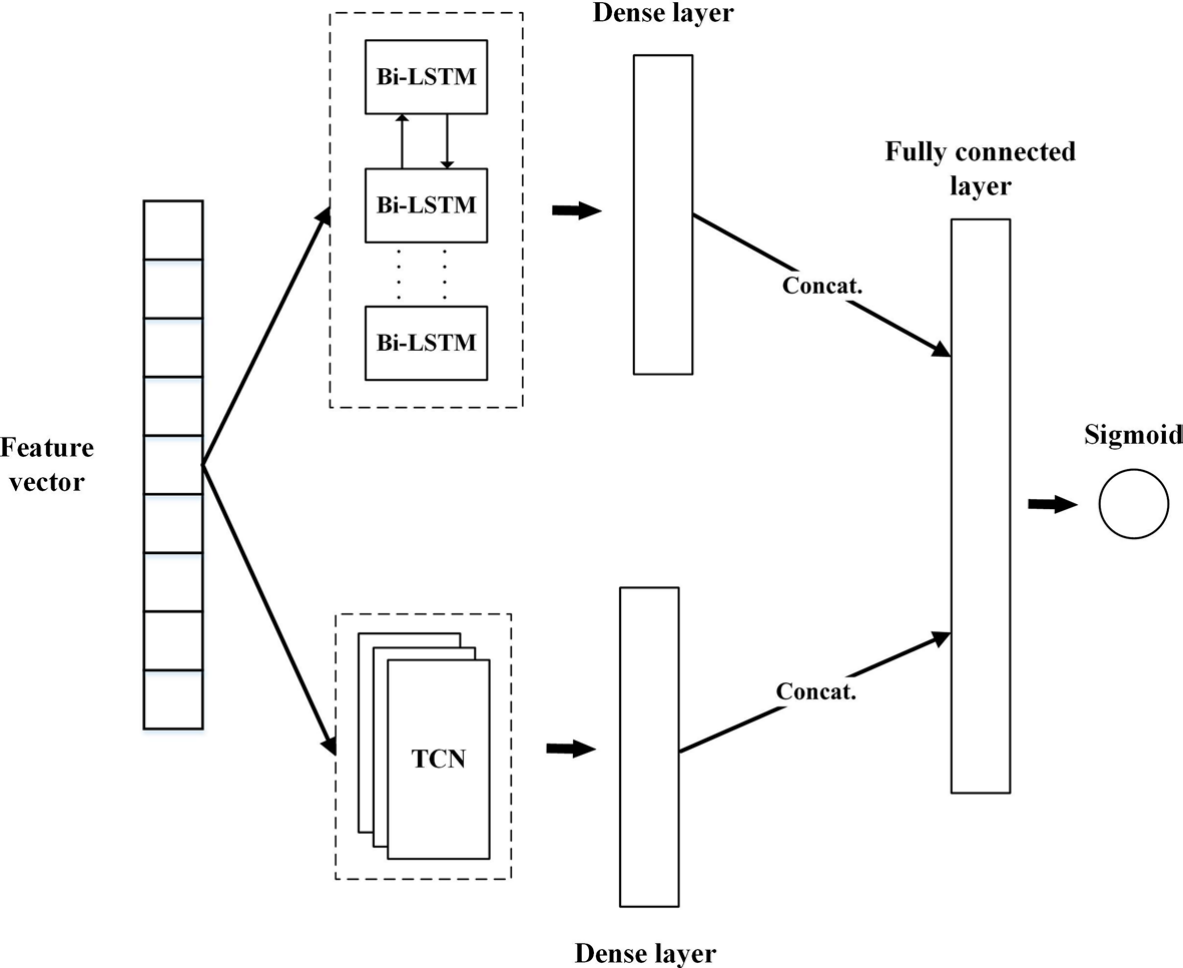
The proposed classifier model

The detail of the proposed classifier model in this paper is shown in Fig. 4. After the feature vector from the text representation is obtained, the vector becomes the input to the Bi-LSTM and TCN cells. Here, Bi-LSTM can remember the forward and backward information of the sequence, while TCN can catch and learn local information sufficiently. Each output of Bi-LSTM and TCN is followed by a dense layer to give the same vector dimensionality. The outputs of both deep learning models are concatenated to become a fully connected layer. The last step of this stage introduces the sigmoid as the activation function due to its fast convergence.

#### Bidirectional long-short term memory

**Fig. 5:**
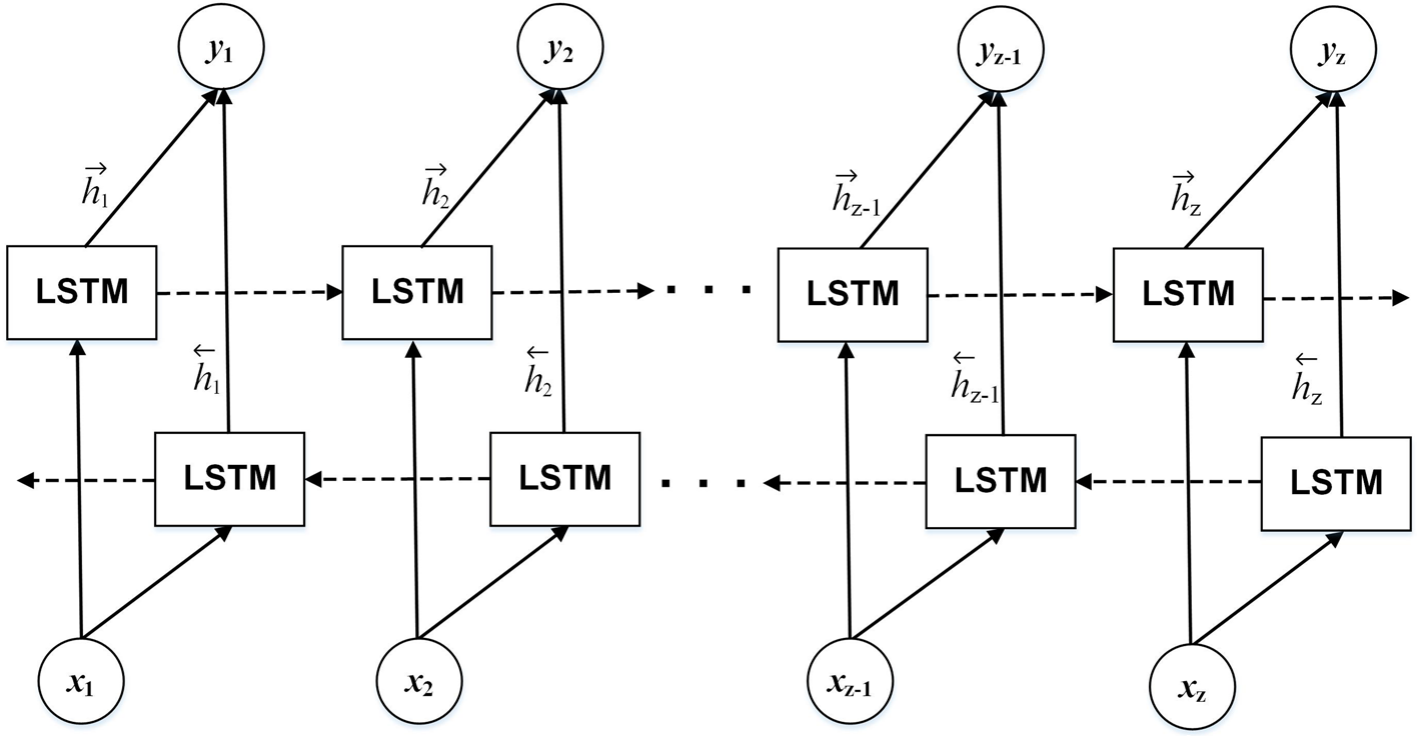
The Bi-LSTM structure

Bidirectional long-short term memory (Bi-LSTM) is an extension of the standard LSTM model that learns the sequence of data in both the forward and backward directions [32]. Thus, the feature vector from the contextualized word embedding can be grasped deeper by considering information from the past and future. Figure 5 denotes the structure of the Bi-LSTM cells. The basic architecture is precisely the same as the standard LSTM, yet the final output is gained by concatenating the forward and backward cells. The final Bi-LSTM output is obtained using Eq. 3, which combines the hidden vector $\mathop{h}\limits^{\leftarrow} _{i}$ of the backward LSTM cell and the hidden vector $\vec{h}_{i}$ of the forward LSTM cell:

1 $$\mathop{h}\limits^{\leftarrow} _{i} = LSTM\left( {x_{i} ,\mathop{h}\limits^{\leftarrow} _{i - 1} } \right),$$

2 $$\vec{h}_{i} = LSTM\left( {x_{i} ,\vec{h}_{i - 1} } \right),$$

3 $$y_{i} = f\left( {\mathop{w}\limits^{\leftarrow} _{i} \mathop{h}\limits^{\leftarrow} _{i} + \vec{w}_{i} \vec{h}_{i} } \right),$$

where $y_{i}$ is the final output Bi-LSTM at the *i*th cell, while $\mathop{w}\limits^{\leftarrow} _{i}$ and $\vec{w}_{i}$ are the output weights of the backward and forward LSTMs at the *i*th cell.

#### Temporal convolutional network

**Fig. 6:**
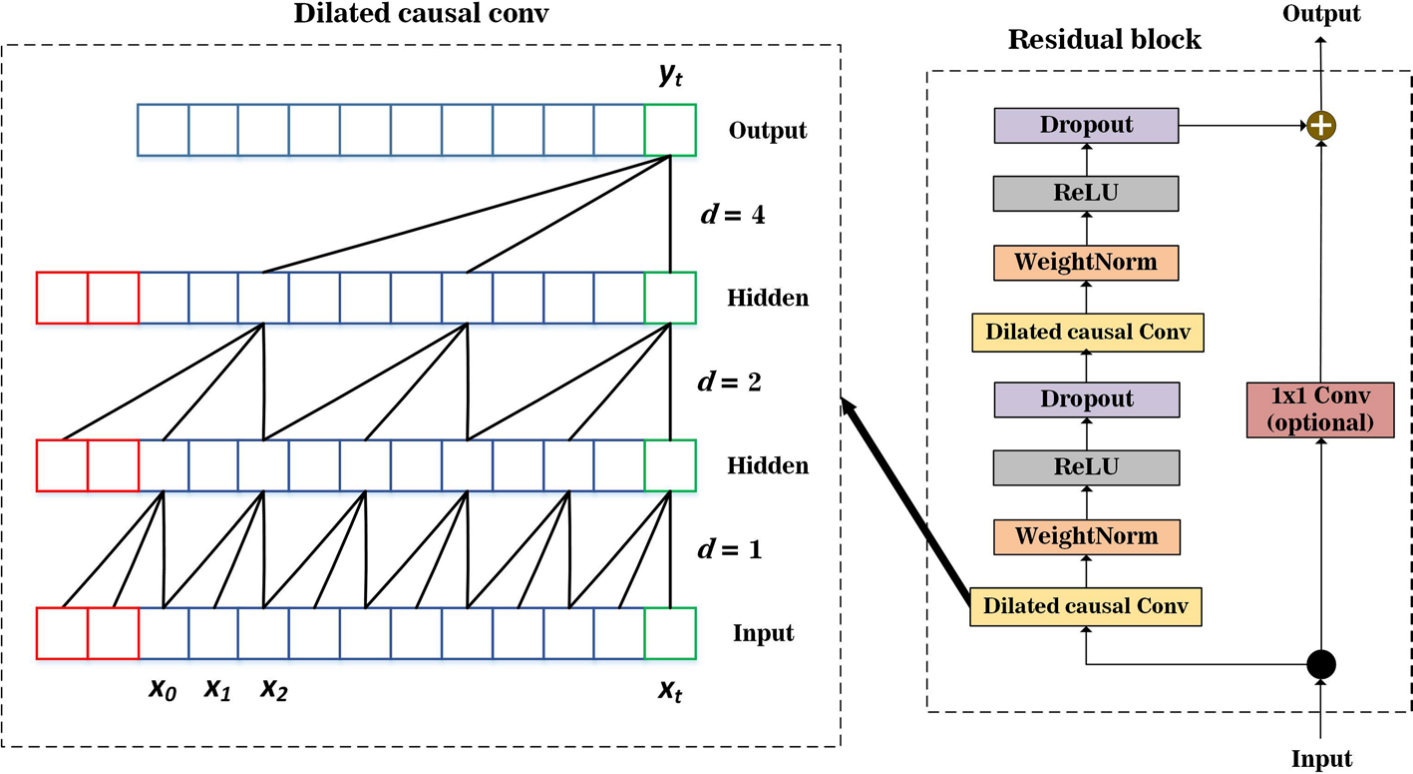
TCN architecture with a filter size of 3

A temporal convolutional network (TCN) exploits the work of one-dimensional convolutional neural networks (1D-CNN) through several modifications. TCN can capture historic dependencies and avoid the loss of long-term information [33]. In contrast with the standard 1D-CNN technique, TCN consists of a number of residual blocks containing dilated causal convolutional layers, weight normalizations, and dropout regularizations, as shown in Fig. 6. The causal convolutional process takes all previous data before time *t* at the previous layer as inputs to predict a certain value at time *t*. However, this causal convolutional layer consumes many layers and requires an extensive filter size and other parameters to enlarge the receptive fields. Hence, the TCN model introduces a dilated approach to obtain a large receptive field. This convolves the input sequences by striding over fixed-step *d* at the previous layer with a filter size, as shown on the left side of Fig. 6. Therefore, the TCN architecture has superiority in memory saving and running speed.

Weight normalization and dropout also are assembled in the architecture after the dilated causal convolution to prevent the overfitting model. The final output of each residual block is connected to the input with a 1 × 1 convolution operation. This 1 × 1 convolution can adjust the tensor shape by reducing dimensionality.

### Weighted ensemble

As shown in Fig. 2, the output of each section in the classifier model, i.e., Bi-LSTM and TCN, is not the final output of the sentiment classification. The next step introduces the weighted ensemble in the proposed strategy to obtain a final conscientious sentiment prediction. The detailed process of the weighted ensemble strategy is shown in Algorithm 1. The prediction results of two text representations from BERT and DistilBERT are integrated using the weighted ensemble strategy to estimate the final sentiment class prediction. We utilized an optimization algorithm to find *μ* weight as a weighted value for the BERT text representation output (*bv*). Then, the λ weighted value for the DistilBERT text representation output (*dv*) is obtained by subtracting 1 from the value of *μ*. After multiplying *bv* and *dv* by their weights, respectively, we take their summative value to obtain the final prediction.


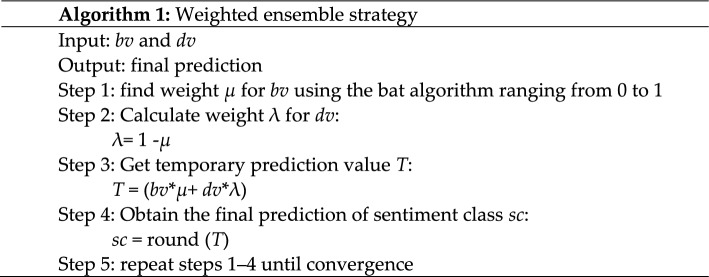


The optimization algorithm used in the weighted ensemble was the bat algorithm proposed by Yang [34]. This bat-inspired optimization method possesses several parameters that provide an efficient outcome, such as frequency settings, automated zooming, and adjusted parameters [35]. The pseudo-code of the bat algorithm is shown below as Algorithm 2 [36].


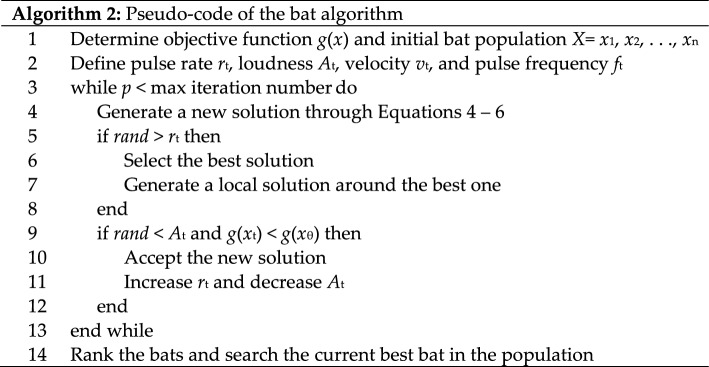


The objective function *g*(*x*) for this problem is the error of the final prediction of sentiment class. The initial values for all parameters can be random values. We take random numbers in [0.0, 1.0] for initial values in pulse rate *r*_t_, loudness *A*_t_, velocity *v*_t_, and pulse frequency *f*_t_. When bats swarm to find their food, they need to update their position, frequency, and velocity. Hence, we calculate the following equations:

4 $$g_{t} = g_{min} + \left( {g_{min} - g_{max} } \right)\beta ,$$

5 $$v_{t}^{p} = v_{t}^{p - 1} + \left[ {x_{t}^{p - 1} - x_{\theta } } \right]g_{t} ,$$

6 $$x_{t}^{p} = x_{t}^{p - 1} + v_{t}^{p} ,$$

where *β* refers to a random number obtained in [0,1] and $x_{{\uptheta }}$ is the best solution of a bat in the swarm at the *p*-th iteration. $v_{t}^{p}$ and $x_{t}^{p}$ are the velocity and position of bat *t* at period step *p.* To prevent the local optimum trap, a new solution around the best current solution is proposed using a random walk:

7 $$x_{new} = x_{old} + \varepsilon A^{p} ,$$

where $\varepsilon$ is a random number obtained in [− 1,1], and $A^{p}$ is the average loudness of the bats at the *p*-th iteration*.* If the condition in line 9 of Algorithm 2 is fulfilled, the bat algorithm reduces the loudness and increases the pulse rate using following formulas:

8 $$A_{t}^{p + 1} = \sigma A_{t}^{p + 1} ,$$

9 $$r_{t}^{p + 1} = r_{t}^{0} \left[ {1 - \exp \left( { - \gamma p} \right)} \right],$$

where σ and γ are constant values, such as the cooling factor in simulated annealing (SA) [37].

## Experiments and results

### Data description

In this sentiment analysis study, we used datasets on various topics in the Indonesian language, and most of the data consisted of short sentences. This study investigated the sentiment on a number of topics, such as cyberbullying, data breaches, regional elections, and others. The data sources came from several platforms, such as Instagram, Twitter, and travel websites. All datasets in this study were already annotated. The detailed information of the experiment datasets is shown in Table 3. After collecting these datasets, this study performed data pre-processing, as explained in the previous section. Then, we randomly mixed all datasets to be trained with the proposed model. In this study, we analyzed the sentiment according to two classes, i.e., positive and negative sentiment. After we performed preprocessing stage, we found that the used datasets are 14,411 samples with a positive sentiment class of 66.89% and a negative sentiment class of 33.11%. Since the available data was imbalanced, we utilized an undersampling scheme to have more balanced data. The undersampling scheme re-samples the dataset by deleting samples from the overrepresented class. A study by [38] examined an undersampling technique to address imbalanced structured data in a binary classification, the balanced accuracy of the undersampling technique outperforms the SMOTE (synthetic minority over-sampling technique) and oversampling techniques in hybrid schemes with lower processing time.

**Table 3:** Dataset information

No.	Topic	Platform source	Number of samples
1	Indonesian 2013 curriculum	Twitter	710
2	Cyberbullying	Instagram	400
3	TV talk show	Twitter	400
4	Cellular service provider	Twitter	300
5	Film review	Twitter	200
6	Head region election	Twitter	900
7	Tourist attraction	Travel website	200
8	Data breach incident	Twitter	1060
9	Others	Twitter	10,805
Total			14,975

### Experiment setting

We conducted and simulated all models in this study using the Python programming language, and the libraries applied in this study were taken from Keras and TensorFlow. The comparison of the number of data between the training data and testing data was 0.8 and 0.2. We configured the parameters for the experiment during the training process in the proposed model using an epoch size of 10 and a batch size of 10 after considering the computation time. As this study focused on two sentiment classes, we selected binary cross-entropy as the loss function for the proposed model. Since we take the BERT Large model, the input dimension for Bi-LSTM and TCN has a size of 1024. For the parameter setting in Bi-LSTM, we set the cell number at 32. For the TCN model, since the text sequence in the input did not have high dimensionality, we set the dilation factor as *d* = 1, 2, 4, 8; the kernel size as *k* = 3; and the filter number as 32. Then, the output space for Bi-LSTM and TCN has a dimensionality size of 32. We also added a dropout for the network. Based on the best result of the experiment, we set the dropout for Bi-LSTM with a ratio of 0.05 and TCN with a ratio of 0.02. As we can see in Fig. 4, the outputs of Bi-LSTM and TCN are fed to a single dense layer. Here, the dense layer has a size of 32 units with ReLU as the activation function.

### Experiment results

To evaluate the proposed model, we take several scheme concepts of related models as counterpart schemes to be adopted and implemented with the same dataset in this study. These included various text representations and classifier models, a brief review of the counterpart scheme concepts is listed below:

- BERT + LSTM-CNN-SVM [9]: this model utilizes a combination of LSTM-CNN-SVM to improve the prediction accuracy in sentiment classification. BERT was selected for text representation.
- BERT and feature union [18]: the BERT base model was chosen by the authors to get the feature vectors from the input texts. A hybrid model of Bi-LSTM and CNN was introduced to better understand the local and context features.
- BERT + LSTM-CNN fusion model [17]: this study made predictions using BERT to extract contextual words. To improve the accuracy, the authors proposed a fusion of LSTM and CNN that could acquire contextual and global information.
- BERT-large + Bi-GRU [14]: the BERT-large model was used as the sentence encoder. Then, Bi-GRU was taken in the model design at the downstream network to get the final classification of each review.
- RoBERTa-LSTM [15]: RoBERTa, as the variant of BERT, was selected for contextualized word embedding. Then, the model can understand contextual semantics dependencies through the LSTM model.
- RoBERTa-CNN [16]: a classification was conducted by introducing a pre-trained text representation model and a classifier model. After performing classification, the combination of RoBERTa as the embedding model and CNN as the classifier model had a satisfactory result.
- Roberta + Bi-GRU-ATT [20]: the authors selected RoBERTa to improve the representation of input texts. Bi-GRU networks were applied after text representation to extract important features. Then, the attention mechanism was presented after Bi-GRU cells to give focus on important features in the sentences.
- SBERT-MLP [13]: this model adopted several text representations and classifier models for spam classification. Based on the accuracy metric in the experiment stage, the hybrid of SBERT (sentence-BERT) and MLP (multilayer perceptron) achieved the highest values in the accuracy metric.

The counterpart models contained several different text representations, such as BERT, RoBERTa, and others. To enhance the sentiment classification performance, various networks were incorporated, such as CNN, LSTM, and others. We first ran the proposed model in the prepared dataset. Then, the counterpart models were trained on the dataset to evaluate the proposed model performance. The overall comparison of the results between the proposed model and the counterpart models is shown in Table 4. We employed several metrics as evaluation indicators, such as accuracy, precision, recall, F-score, and AUC (area under the curve).

**Table 4:** Comparison between the proposed model and the counterpart models

Model	Accuracy*	Precision*	Recall*	F-score*	AUC*
BERT + LSTM-CNN-SVM	75.50	73.73	77.10	74.63	76.33
BERT and feature union	82.60	82.23	82.83	82.50	82.67
BERT + LSTM-CNN fusion model	83.50	82.23	84.83	83.17	83.77
BERT-large + BiGRU	84.60	86.23	83.60	84.83	84.77
RoBERTa-LSTM	83.30	82.17	84.10	83.07	83.33
RoBERTa-CNN	83.00	82.77	83.37	82.93	83.23
Roberta + BiGRU-ATT	82.87	81.40	84.03	82.57	83.03
SBERT-MLP	80.83	82.53	78.60	80.30	81.17
Proposed model	85.13	85.41	84.94	85.17	85.14

*Metric values are expressed as percentages

As shown in Table 4, a single BERT text representation could achieve a significant result when it was combined with Bi-GRU, obtaining an accuracy value of 84.60, a precision value of 86.23, an F-score value of 84.83, and an AUC value of 84.77. The bidirectional forward and backward learning of Bi-GRU (as the classifier model) distinctly contributed to extract the feature information. For the RoBERTa text representation, the best performance was obtained by the combination of RoBERTa model with LSTM, achieving an accuracy value of 83.30, a recall value of 84.10, an F-score value of 83.07, and an AUC value of 83.33. The LSTM model provides an understanding of the sequence information dependencies in the input vectors.

However, our proposed model was the best scheme, as it outperformed all counterpart models in the evaluation metrics. This result was indicated by achieving the highest values in the accuracy metric, with a value of 85.13, a recall metric of 84.94, an F-score metric of 85.17, and an AUC value of 85.14. Regarding the accuracy metric, the proposed model was the only model to exceed a score of 85. This also occurred in F-score and AUC metrics. The F-score measured the harmonic mean of the precision and recall values, while the AUC metric indicated the proficiency of the model in differentiating the sentiment classes and is commonly used in binary classification tasks. The proposed model outperformed the second-best model, a hybrid model of BERT-large and Bi-GRU, despite not being prominen. This may have been caused by this study mixing datasets with various topics and the small size of the Indonesian dataset. However, the performance of the proposed model in this study generally is the best model.

The excellent result of the proposed model was influenced by the combination of BERT and DistilBERT in the text representation section. In addition, Bi-LSTM provides crucial information on the sequence features of the texts to grasp the contextual understanding, while TCN extracts the local spatial–temporal features to obtain the semantic information. In the counterpart models, several schemes utilized the LSTM model; however, they did not employ the bidirectional scheme of LSTM, as in the proposed model. Therefore, the TCN model combined with Bi-LSTM proved a better understanding of the contextual and semantics features of text sequences.

**Fig. 7:**
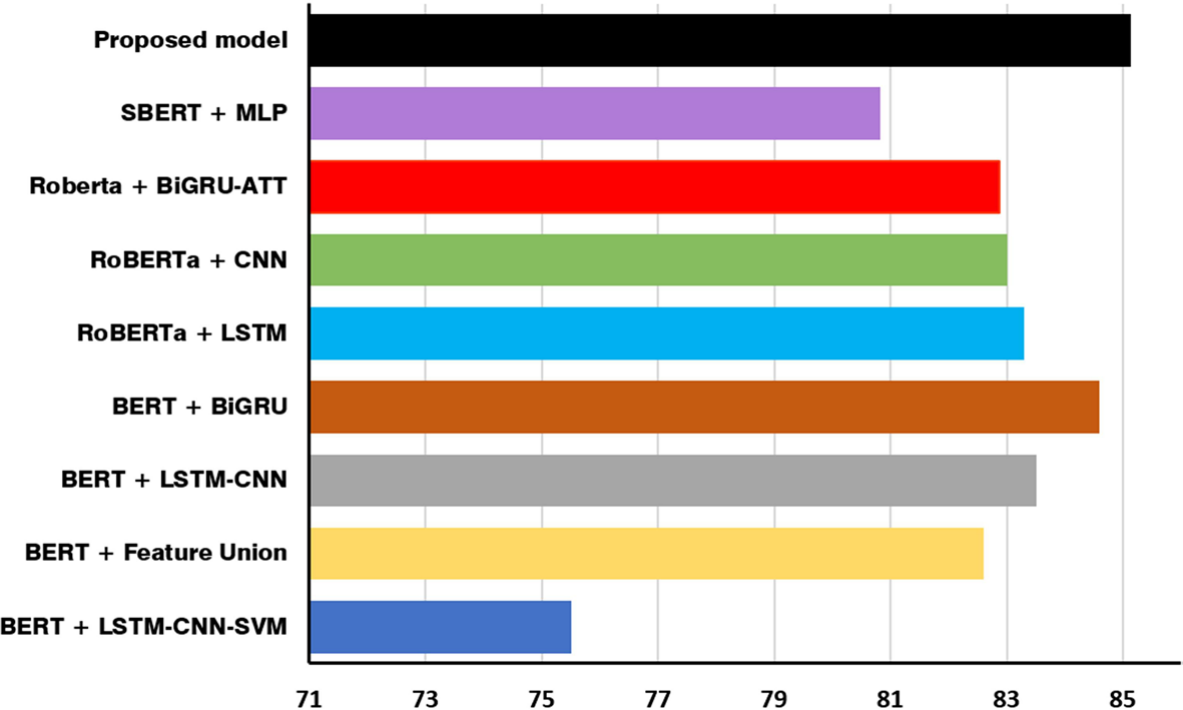
Comparison of the accuracy metric as a percentage

In addition, we presented the accuracy values as a common metric to show the comparison of all models from a graphic perspective, as shown in Fig. 7. The figure shows that the proposed model was the only scheme to exceed an accuracy value of 85.00. The only model to perform close to the proposed model was a hybrid model of BERT and Bi-GRU. Based on this figure, we can see that the single text representation using BERT fell behind the hybrid model of BERT and DistilBERT in our proposed model. In addition, the bidirectional learning of its recurrent neural network was not sufficient to extract the semantic and contextual features of the texts. Various studies have indicated that hybrid approaches in deep learning tasks can present matching or superior results compared to single approaches [9]. In general, the hybrid model in this study performed better than any single model in text representation and classification model.

Most of the above counterpart models utilize a hybrid or ensemble approach in the classifier part of the architecture. To know the performance of the hybrid model on the text representation part, we presented a study that proposed an ensemble model using the combination of BERT and RoBERTa [39], to be compared with our proposed model. This strategy took the pooler output from the pre-trained models, i.e., BERT and RoBERTa, and then concatenated the outputs going to the LSTM cells and an extra attention layer before the final classification. However, this work [39] only performed a simple hybrid approach by concatenating the output of each text representation to become inputs for the classifier section. It did not propose the hybrid approach in the classifier section. We then compared the precedent work with the proposed model, as shown in Table 5. In this Table, we also presented the results of the single text representation scheme of BERT and DistilBERT combined with Bi-LSTM and TCN but did not apply the weighted ensemble as our proposed model.

**Table 5:** Evaluation of text representation ensemble

Model	Accuracy*	Precision*	Recall*	F-score*	AUC*
BERT and RoBERTa + LSTM-attention [34]	82.80	79.70	85.07	82.21	83.05
BERT + BiLSTM-TCN	84.65	84.67	84.66	84.65	84.68
DistilBERT + BiLSTM-TCN	82.28	84.33	81.09	82.62	82.44
Our Proposed model	85.13	85.41	84.94	85.17	85.14

*Metric values are expressed as a percentage

Based on the metric evaluation, the counterpart models could not outperform the proposed model. The proposed model only failed in the recall metric with a slight difference. The simple method in the ensemble strategy was not adequate for enhancing the classification task performance. In addition, the classifier model had a crucial impact on the final prediction accuracy. It was critical for the classifier model to understand the context and semantics of the sentences. Afterward, Table 5 also shows that single text representation using BERT or DistilBERT without implementing the weighted ensemble does not achieve a prominent result. The proposed model attained excellent results by incorporating the weighted ensemble for the text representation and the hybrid scheme in the classifier model.

## Conclusion and future work

This study introduced a hybrid deep learning model approach for sentiment analysis of Indonesian-language text. This study did not focus only on one topic for sentiment study, but rather conducted sentiment analysis on various mixed topics from several platforms. Then, this study presented the combination of the text representation of BERT and DistilBERT to gain optimal text representation. To obtain the important semantic and contextual features of the texts, we also proposed a hybrid model of Bi-LSTM and TCN. Introducing the weighted ensemble to obtain the final prediction of two schemes provided a significant improvement in sentiment classification. The experimental results indicated that the proposed model attained high reliability in performing sentiment analysis conducted on various Indonesian topics. The results also indicated the metric evaluation of the proposed model outperformed those of the counterpart models.

In future work, our study may focus on investigating how to augment datasets. Since the availability of labeled Indonesian datasets is low compared to those in English, it is crucial to generate a synthetic dataset that has similarities to the real data. Synthetic data generation can be performed in deep generative models through adversarial training or recurrent neural network models. When the availability of training data is larger, the model can achieve better performance result that is superior to current methods.

## Data Availability

The data for this experiment are freely available to the public via links https://github.com/cahkanor/id-kurikulum2013-sentimentanalysis, https://github.com/rizalespe/Dataset-Sentimen-Analisis-Bahasa-Indonesia, https://github.com/cahkanor/id-tourism-sentimentanalysis, https://github.com/nadhifikbarw/ep-dataset, and https://ridi.staff.ugm.ac.id/2019/03/06/indonesia-sentiment-analysis-dataset.

## Abbreviations

NLP: Natural language processing
BERT: Bidirectional encoder representations from transformers
RoBERTa: Robustly optimized BERT pre-training approach
LSTM: Long short-term memory
CNN: Convolutional neural network
SVM: Support vector machines
Bi-LSTM: Bidirectional long short-term memory
TCN: Temporal convolutional networks
TF-IDF: Term frequency–inverse document frequency
SMS: Short message service
BoW: Bag-of-words
MLP: Multilayer perceptron
Bi-GRU: Bidirectional gate recurrent unit
SGD: Stochastic gradient descent
MLM: Masked-language modeling
NSP: Next sentence prediction
SMOTE: Synthetic minority over-sampling technique
SBERT: Sentence-BERT
MLP: Multilayer perceptron
AUC: Area under the curve
